# Effects of PVP/NVP Additives on the Surface Wettability and Hydration Kinetics of Low-Silicone TRISS-Based Hydrogel Contact Lenses

**DOI:** 10.3390/gels12040276

**Published:** 2026-03-26

**Authors:** Jaehyeung Kim, Sangjun Pyo, Hyerin Ahn, Ok Chan Jeong

**Affiliations:** 1 Department of Digital Anti-Aging Health Care, Graduate School of Inje University, Gimhae 50834, Republic of Korea; kyj85202@naver.com (J.K.); dumack086@naver.com (S.P.); gpfls5653@naver.com (H.A.); 2 Department of Biomedical Engineering, College of Biomedical Science & Health, Inje University, Gimhae 50834, Republic of Korea

**Keywords:** silicone hydrogel, 3-[tris(trimethylsilyloxy)silyl]propyl methacrylate (TRISS), polyvinylpyrrolidone (PVP), N-vinyl-2-pyrrolidone (NVP), oxygen transport, surface wettability, hydration-induced dimensional stability

## Abstract

Silicone hydrogels offer high oxygen permeability but suffer from poor wettability. This study integrates a TRISS-based system (0–2.0 wt%) with a fixed PVP/NVP matrix (1.0/0.5 wt%) to enhance hydration-induced dimensional stability and surface properties. Fabricated via cast-molding, the lenses demonstrated that TRISS incorporation significantly enhances oxygen transport. Specifically, the 2.0 wt% TRISS formulation (S2.0) achieved an ~1.9-fold increase in oxygen-induced current (from 0.97 μA in pure-HEMA to 1.86 μA) while strongly suppressing hydration-induced swelling. To counter TRISS’s inherent hydrophobicity, the PVP/NVP matrix acted as a vital compensatory mechanism, driving the equilibrium contact angle down to 56.04° and avoiding the severe hydrophobic plateau (93.79°) of the additive-free comparator. S2.0 maintained a robust oxygen response alongside improved wettability. In conclusion, this system defines a workable low-silicone design window accommodating up to 2.0 wt% TRISS without wettability loss or optical degradation (>97%). Crucially, by leveraging TRISS to mitigate swelling-induced mechanical stress and PVP/NVP to ensure stable wettability, this structurally robust hydrogel provides a highly viable foundational matrix for future smart contact lenses equipped with diagnostic micro-components.

## 1. Introduction

In the modern digital era, the prevalence of eye diseases such as dry eye syndrome has increased significantly due to the prolonged use of electronic devices [1]. Dry eye syndrome is defined as a multifactorial disease of the ocular surface characterized by a loss of tear film homeostasis. It manifests through symptoms where tear film instability, hyperosmotic pressure, ocular surface inflammation, and neurosensory abnormalities play critical roles in its etiology [2]. Notably, contact lens users experience dry eye symptoms 12 times more frequently than emmetropic individuals and 5 times more frequently than spectacle wearers. A mechanistic explanation for this higher prevalence is that the tear film thins more rapidly due to evaporation and dehydration, subsequently increasing the osmotic pressure of the tear film [3]. Other contributing factors include the use of high-water-content lenses, which, despite providing initial comfort, are often associated with spoilation and deposition that can compromise long-term wearing comfort [3]. Although soft contact lenses offer convenient vision correction, their high water content often causes the eye surface to become dehydrated over time, leading to discomfort and structural deformation. This dehydration disrupts the balance of the tear film, exacerbating dry eye syndrome; furthermore, prolonged wear accelerates tear evaporation, causing further dryness and limiting tear retention capacity [4,5,6,7].

Conventional hydrogel-based contact lenses are favored for their high water content, which offers immediate moisture. However, these lenses often undergo repeated hydration and dehydration cycles during wear, reducing their morphological stability and mechanical strength over time [5,8]. This degradation impairs tear film stability, leading to ocular fatigue and exacerbating dry eye symptoms. Furthermore, limited oxygen transport in conventional hydrogels can lead to ocular surface hypoxia, an environment where increased reactive oxygen species (ROS) can potentially harm ocular tissues [9,10]. Consequently, key performance parameters [11] for next-generation lenses must include balanced light transmittance [12], morphological stability [5], water content and wettability [7], oxygen transport [13], and mechanical strength [14].

Recent evaluations have further emphasized the importance of maintaining optical clarity and dimensional stability under varying environmental conditions to ensure consistent visual performance. In particular, the strategic integration of internal wetting agents has emerged as a primary strategy to mitigate the trade-offs between oxygen delivery and surface hydration in advanced silicone-based materials. Water content is essential for maintaining comfort; however, excessive water content accelerates moisture loss in dry environments, leading to lens dehydration and reduced wearing comfort [6,7]. Additionally, changes in lens diameter caused by hydration affect both the wearing comfort and the structural stability of the lens [5,15]. Oxygen transport is equally vital because the cornea is avascular and depends on oxygen from the external environment. Limited oxygen transmission leads to corneal swelling and increases the risk of long-term complications [9,10].

To address these physiological challenges, silicone-based monomers such as 3-[tris(trimethylsilyloxy)silyl]propyl methacrylate (TRISS) have been extensively explored. These materials offer enhanced oxygen transport potential and improved structural integrity compared to conventional hydrogel lenses [16]. However, the inherent hydrophobicity of the siloxane groups in TRISS significantly limits the equilibrium water content and surface wettability [16,17,18], which can lead to lipid deposition and reduced clinical comfort. Previous studies have indicated that while TRISS-based formulations enhance oxygenation and mechanical stability, their clinical application is often limited by reduced surface hydration [16,19]. To mitigate this hydrophobicity, hydrophilic additives such as polyvinylpyrrolidone (PVP) and N-vinyl-2-pyrrolidone (NVP) have been incorporated into these systems [17,18] based on an internal wetting agent strategy. Within this strategic design, high-molecular-weight PVP acts as a humectant to entrap water, though it can be prone to phase separation in a hydrophobic siloxane matrix. Conversely, NVP acts as a critical monomeric ‘anchor’ and co-solvent, physically binding the PVP within the network to prevent leaching [17,18].

This study, therefore, aims to systematically investigate the effects of TRISS concentration and the presence of a fixed PVP/NVP matrix on the fundamental properties of contact lenses. By analyzing these designed variables up to a targeted limit of 2.0 wt% TRISS—defined here as the hydrophobic threshold within the present formulation window—we sought to identify a workable low-silicone design window. Literature confirms that siloxane content exceeding 2–3 wt% in conventional HEMA systems can trigger severe macro-phase separation, exponential protein adsorption, and optical deterioration [16,17]. Accordingly, 2.0 wt% was selected as the upper bound examined in this study to improve the oxygen-transport response without crossing into a catastrophic hydrophobic plateau. These findings are expected to provide a robust technical framework for overcoming the limitations of conventional hydrogels and advancing next-generation contact lens technology, particularly for smart contact lens applications involving ocular drug delivery [20], microfluidic tear-analysis [21], theranostic applications [22], tear-glucose sensing [23], and intraocular pressure monitoring [24]. While these emerging directions support the growing role of soft contact lenses as wearable platforms for health management [25,26], their clinical translation faces severe material-level bottlenecks. Specifically, as highlighted in a recent critical review by Han et al. [27], while silicone-based substrates offer essential oxygen permeability, their inherent hydrophobicity and susceptibility to biofouling pose major hurdles for continuous physiological monitoring, strongly necessitating the strategic integration of hydrophilic monomers. Furthermore, another primary challenge is the mechanical mismatch at the interface of rigid microelectronic sensors and soft hydrogel substrates; excessive volume expansion (swelling) of conventional hydrogels upon hydration causes shear stress that leads to sensor delamination [23,26]. Additionally, when surface microstructures (e.g., microfluidic channels) are integrated, unpredictable hydration swelling severely distorts their pre-designed dimensions, leading to the functional failure of the fluidic dynamics [21].

Therefore, establishing a structurally robust substrate with strictly controlled dimensional stability and improved wettability is urgently needed. By successfully suppressing hydration-induced expansion (maintaining an optimal ~15% maximum diameter change) and effectively overcoming the hydrophobic persistence of the siloxane groups through our fixed dual-hydrophilic PVP/NVP matrix, the TRISS-PVP/NVP hydrogel platform proposed in this study directly addresses the exact engineering challenges emphasized by Han et al. [27] and others. It effectively mitigates both interfacial shear stress for rigid components and geometric warpage for micro-structures, providing a highly stable and viable material foundation for the diagnostic lenses of tomorrow.

## 2. Results and Discussion

To evaluate the influence of the silicone monomer (TRISS) and the dual-hydrophilic matrix (PVP/NVP) on lens performance, ten distinct formulations were prepared and categorized into the S-series and the E-series. To maintain a systematic comparison, two distinct control groups were established: (1) Pure-H, which serves as the absolute pHEMA reference free of TRISS or PVP/NVP, and (2) S0.0, which acts as the internal baseline for the S-series to evaluate the specific influence of TRISS within the fixed PVP/NVP matrix. The sample nomenclature and detailed chemical compositions are summarized in Table 1. In this study, the TRISS concentration and the presence of the PVP/NVP matrix were the primary designed formulation variables. While auxiliary components such as EGDMA, GMA, MA, and ADVN were maintained within narrow normalized ranges across the formulation set (EGDMA, 0.656–0.691 wt%; GMA, 0.188–0.198 wt%; MA, 0.028–0.030 wt%; ADVN, 0.300–0.316 wt%), they were not treated as independent design variables; therefore, the observed formulation-dependent differences were interpreted primarily in relation to TRISS concentration and the presence of the PVP/NVP matrix.

### 2.1. Light Transmittance

**Figure 1:**
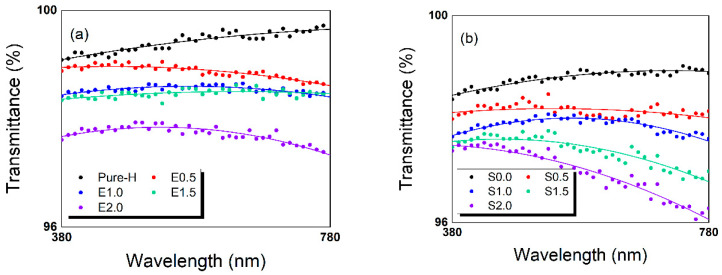
Light transmittance spectra of the fabricated contact lenses across the visible spectrum (380–780 nm). (**a**) Non-additive counterparts (E-series) containing varying TRISS concentrations, (**b**) PVP/NVP-containing S-series incorporating the PVP/NVP hydrophilic matrix. In each panel, the differently colored lines represent the individual lens formulations indicated in the legend.

Figure 1 and Table A1 demonstrate that all lens specimens, including those with TRISS and hydrophilic additives, maintained high visible light transmittance exceeding 97%. These results fully satisfy the international optical requirements for contact lens applications. In the additive-free Group 1, increasing the TRISS content from 0% (Pure-H) to 2.0% (E2.0) resulted in a marginal decrease in transmittance across the visible spectrum (380–780 nm). A similar trend was observed in the PVP/NVP-containing S-series (Group 2), where the transmittance slightly decreased as silicone content increased.

This phenomenon is attributed to the increased silicone domains within the hydrogel matrix, which can induce minor light scattering, particularly when integrated with hydrophilic regions. Specifically, the absolute control lens (Pure-H) exhibited an average transmittance of ~99.3%, while the 2.0 wt% TRISS formulation (E2.0) averaged ~97.3%. Within the S-series, the hydrophilic baseline (S0.0) maintained a high transparency of ~98.9%, which slightly adjusted to ~97.3% for the S2.0 formulation. This minimal reduction in optical clarity, consistent with previous literature [12,17], confirms that the incorporation of TRISS and the PVP/NVP additive system at these concentrations preserves the fundamental optical performance of the lenses.

### 2.2. Hydration Kinetics and Hydration-Induced Dimensional Stability

**Figure 2:**
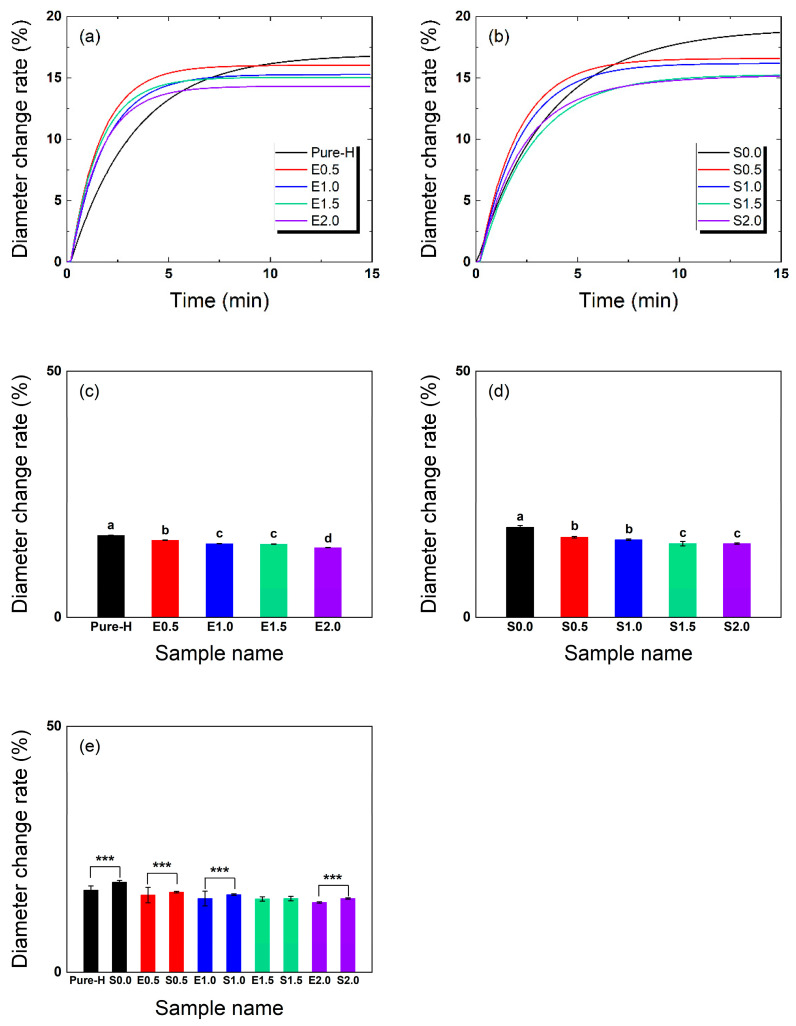
Hydration-induced diameter change over time. (**a**) Time-course diameter expansion for the additive-free E-series, (**b**) Time-course diameter expansion for the PVP/NVP-containing S-series, (**c**) Comparison of maximum diameter change at 15 min for the E-series, (**d**) Comparison of the S-series, (**e**) Statistical comparison between E-series and S-series at identical TRISS concentrations. (*n* = 3. Exact F-statistics and *p*-values for the ANOVA and pairwise comparisons are detailed in the Section 2. Different lowercase letters indicate significant differences among groups according to Tukey’s post hoc test following one-way ANOVA in panels (**c**) and (**d**), and *** indicates *p* < 0.001 for the pairwise comparison in panel (**e**)).

Figure 2 shows the hydration-induced diameter change over time, demonstrating that increasing the TRISS content in the lens reduces swelling upon hydration and thereby improves hydration-induced dimensional stability. The dynamic hydration process was quantitatively evaluated by fitting the diameter-change profiles to the exponential approach-to-equilibrium model described in Section Hydration-Induced Diameter Change, Equation (1), from which the hydration time constant ($\tau_{D}$) was derived.

The maximum diameter change rates for the S-series, which reflect the final expansion at equilibrium, are summarized in Table 2. The diameter change rate exhibited a clear decreasing trend as TRISS concentration increased. After complete hydration, the 2.0 wt% TRISS formulation (S2.0) exhibited a maximum diameter change of 15.1%, whereas the absolute control hydrogel (Pure-H) showed 16.7%. In contrast, the baseline formulation containing exclusively hydrophilic additives (S0.0) exhibited the highest expansion rate of 18.3%.

Comparative analysis at the 15-min mark (Figure 2c,d) confirmed that the incorporation of TRISS is the primary factor suppressing excessive swelling. The significant difference observed between Pure-H and S0.0 (Figure 2e) suggests that hydrophilic additives alone tend to increase swelling by enhancing water uptake.

The diameter change curves also revealed that formulations containing TRISS reached saturation within a specific period, whereas the additive-only (S0.0) and absolute control (Pure-H) formulations required extended durations to achieve full saturation within 15 min. This indicates that TRISS effectively prevents excessive swelling while simultaneously promoting a faster attainment of hydration equilibrium.

Regarding hydration kinetics, the measured data were non-linearly fitted to a time-dependent dynamic process. As summarized in Table 2, the hydration time constants for diameter expansion ($\tau_{D}$) in the S-series ranged from 2.05 to 3.46 min, compared with 3.26 min for Pure-H. At the highest TRISS loading, S2.0 showed a τ_D_ of 2.45 min and a maximum diameter change of 15.15%, indicating controlled hydration-induced expansion within the fixed PVP/NVP matrix. Rather than claiming a dramatic acceleration of hydration kinetics, the present data support that the 2:1 PVP/NVP matrix functions primarily as a compensatory system that mitigates the hydrophobic penalty of TRISS while preserving mechanical flexibility.

**Figure A1:**
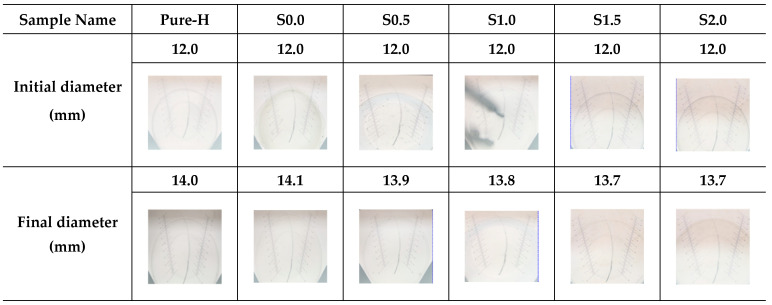
Representative images of lens specimens before and after hydration. Comparison between the initial dry state (12.0 mm diameter) and the fully hydrated equilibrium state (13.7–14.1 mm) for the selected formulations (Pure-H to S2.0). These images illustrate the hydration-induced dimensional response of the S-series, showing limited expansion despite the presence of hydrophilic additives.

ANOVA results (Figure 2c–e) confirmed statistically significant differences in diameter change rates among the groups (E-series: F(4,10) = 889.12, *p* = 1.04 × 10^−12^; S-series: F(4,10) = 5987.10, *p* = 7.60 × 10^−17^). For the pairwise matched comparisons in Figure 2e, the exact *p*-values are: Pure-H vs. S0.0 (*p* = 1.44 × 10^−15^), E0.5 vs. S0.5 (*p* = 3.18 × 10^−9^), E1.0 vs. S1.0 (*p* = 1.68 × 10^−13^), E1.5 vs. S1.5 (*p* = 0.99997, not significant), and E2.0 vs. S2.0 (*p* = 6.38 × 10^−14^). Within the S-series, TRISS-containing formulations (S0.5–S2.0) exhibited significantly lower expansion rates compared with the additive-only baseline (S0.0). These findings demonstrate that TRISS plays a decisive role in enhancing resistance against hydration-induced expansion. Conversely, excessive hydrophilic additives can increase the swelling ratio, underscoring the necessity of calibrating the ratio between silicone monomers and hydrophilic components. Representative images before and after hydration are provided in Figure A1 (Appendix B), further illustrating the expansion characteristics observed in each group.

### 2.3. Water Content

**Figure 3:**
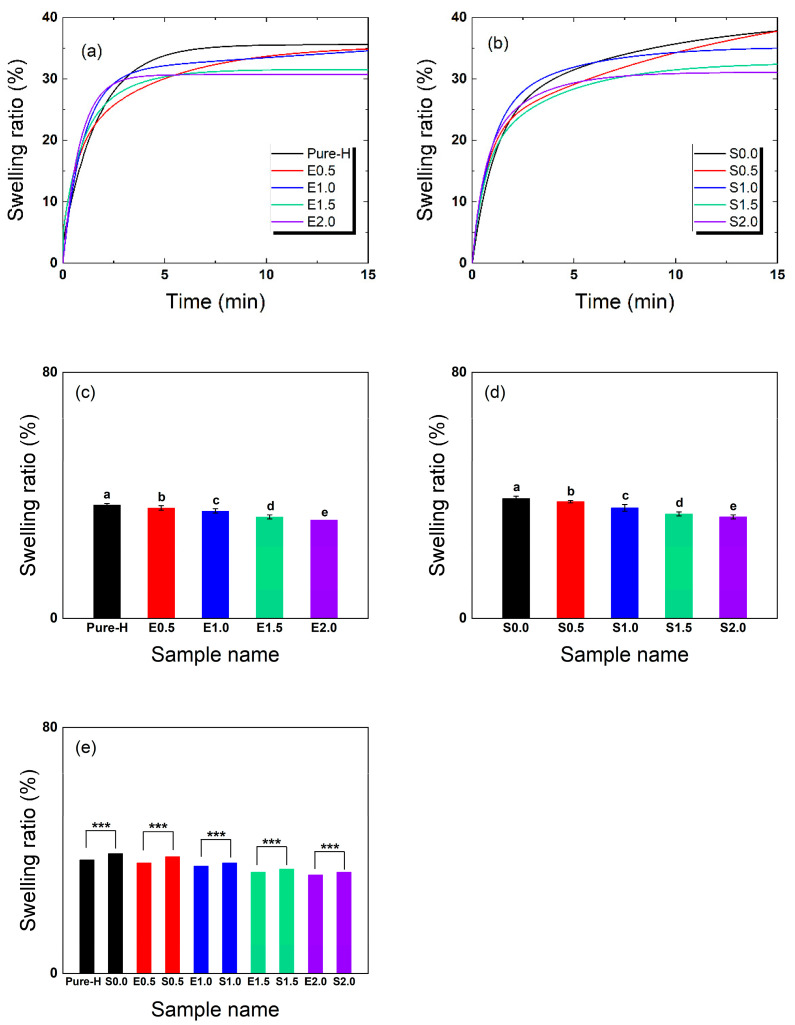
Equilibrium water content (EWC) of the fabricated lenses. (**a**) EWC kinetics for the additive-free E-series, (**b**) EWC kinetics for the PVP/NVP-containing S-series, (**c**,**d**) ANOVA of final EWC values for both groups, (**e**) Comparison of water-retention capability between the additive-free and PVP/NVP-containing series. (*n* = 3. Exact F-statistics and *p*-values for the ANOVA and pairwise comparisons are detailed in the Section 2. Different lowercase letters indicate significant differences among groups according to Tukey’s post hoc test following one-way ANOVA in panels (**c**) and (**d**), and *** indicates *p* < 0.001 for the pairwise comparisons in panel (**e**)).

Figure 3 illustrates the equilibrium water content (EWC) of the contact lenses during hydration. For the additive-free formulations, the EWC ranged from approximately 32% (E2.0) to 37% (Pure-H) under fully hydrated conditions. In the PVP/NVP-containing S-series, the addition of NVP and PVP slightly increased the EWC, with values ranging from approximately 33% to 39%, the highest being ~39% for the S0.0 baseline. The hydration behavior of all specimens followed a pseudo-first-order kinetic model. To quantitatively evaluate the dynamic water uptake, the hydration time constant ($\tau_{EWC}$), which represents the time required to reach approximately 63.2% of the equilibrium water content, was calculated. The resulting kinetic parameters are summarized in Table 2.

The reduced water uptake in formulations with higher TRISS content is expected, as silicone-based hydrogels generally possess lower EWCs than conventional hydrogels due to their hydrophobic composition. This inherent hydrophobicity is also reflected in the surface wettability behavior discussed in Section 2.4. Conversely, the PVP/NVP matrix improved surface wettability and slightly increased water retention, effectively offsetting the hydrophobicity of the silicone.

ANOVA results (Figure 3c–e) confirmed statistically significant differences in equilibrium water content among the groups (E-series: F(4,10) = 16,608.11, *p* = 4.63 × 10^−19^; S-series: F(4,10) = 7382.28, *p* = 2.67 × 10^−17^). For the matched comparisons in Figure 3e, all differences were highly significant: Pure-H vs. S0.0 (*p* = 1.44 × 10^−15^), E0.5 vs. S0.5 (*p* = 1.44 × 10^−15^), E1.0 vs. S1.0 (*p* = 1.67 × 10^−15^), E1.5 vs. S1.5 (*p* = 1.67 × 10^−15^), and E2.0 vs. S2.0 (*p* = 1.67 × 10^−15^). Within the S-series, all TRISS-containing lenses (S0.5–S2.0) exhibited significantly lower swelling ratios compared to the baseline formulation containing exclusively hydrophilic additives (S0.0). A comparative analysis further revealed that while hydrophilic additives alone (as seen in S0.0) increase swelling, the incorporation of TRISS effectively balances this effect. These findings highlight that TRISS is the primary factor in regulating hydration-induced expansion, ensuring material stability even in the presence of hydrophilic additives. The measured EWC values for the S-series were consistent with the overall formulation-dependent trends observed in this study.

### 2.4. Contact Angle (Wettability)

**Figure 4:**
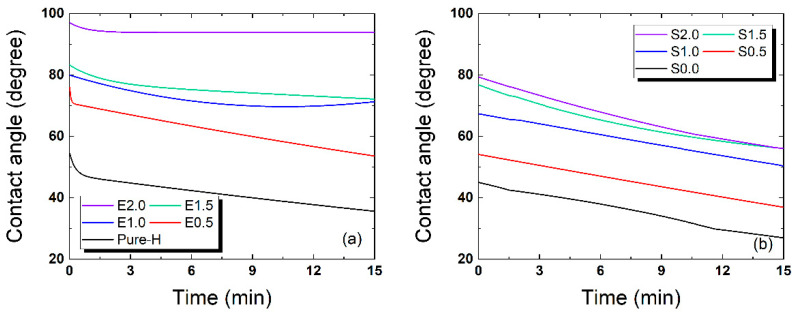
Time-dependent contact angle changes for surface wettability evaluation. (**a**) Contact angle decay for the additive-free E-series, (**b**) Contact angle decay for the PVP/NVP-containing S-series, demonstrating a clear transition to a stable hydrophilic equilibrium.

Figure 4 presents the change in contact angle over time, illustrating the significant variations in surface wettability depending on the TRISS concentration and the presence of hydrophilic additives. The results highlight the opposing effects of hydrophobic siloxane groups and the internal wetting agents. As expected, additive-free formulations with higher TRISS concentrations exhibited significantly higher static contact angles, indicating a transition toward a hydrophobic surface. For the additive-free Group, the initial contact angle increased from 53.92° for Pure-H to 95.42° for E2.0 (2.0 wt% TRISS). This substantial increase demonstrates that TRISS, due to its hydrophobic siloxane groups, severely impairs surface wettability. In contrast, the PVP/NVP-containing S-series formulations exhibited a marked improvement in hydrophilicity.

The initial contact angle for S2.0 (2.0 wt% TRISS with additives) was measured at 78.76°, which is significantly lower than that of its additive-free counterpart, E2.0 (95.42°). Even for the silicone-free baseline, the addition of NVP/PVP reduced the initial contact angle from 53.92° (Pure-H) to 49.17° (S0.0). The dynamic wettability behavior further underscored the efficacy of the PVP/NVP matrix, particularly regarding the stark contrast in the final equilibrium states. After a specific period of water droplet exposure, E2.0 maintained a high contact angle of 93.79°, indicating persistent hydrophobicity and an irreversible stall at a hydrophobic plateau. Conversely, the contact angle for S2.0 decreased to 56.04°, completely bypassing this hydrophobic plateau.

These results clearly demonstrate that NVP and PVP effectively counteract the inherent hydrophobicity introduced by TRISS, facilitating the formation of a more wettable lens surface. This trend aligns with literature suggesting that internal wetting agents like PVP are essential for enhancing surface moisture retention in commercial silicone hydrogel lenses. This study confirms that the benefits of high oxygen transport potential provided by TRISS can be achieved while preserving surface wettability by employing a calibrated PVP/NVP concentration. Furthermore, while these measurements provide valuable comparative insights into material surface properties, we transparently acknowledge the inherent limitations of standard in vitro contact angle tests, which do not reproduce the multifaceted in vivo environment involving proteins, lipids, mucins, and dynamic factors like blinking [23]. Therefore, the time constant ($\tau_{CA}$) serves as an indicator of in vitro surface wetting recovery under specific laboratory conditions, rather than a direct measure of in vivo tear-film stability [28]. The relationship between our measured wetting kinetics and actual pre-lens tear-film stability remains to be confirmed through future clinical studies, such as pre-lens tear film break-up time evaluations and deposition-challenge models [29,30].

### 2.5. Oxygen Transport Evaluation via Oxygen-Induced Current

**Figure 5:**
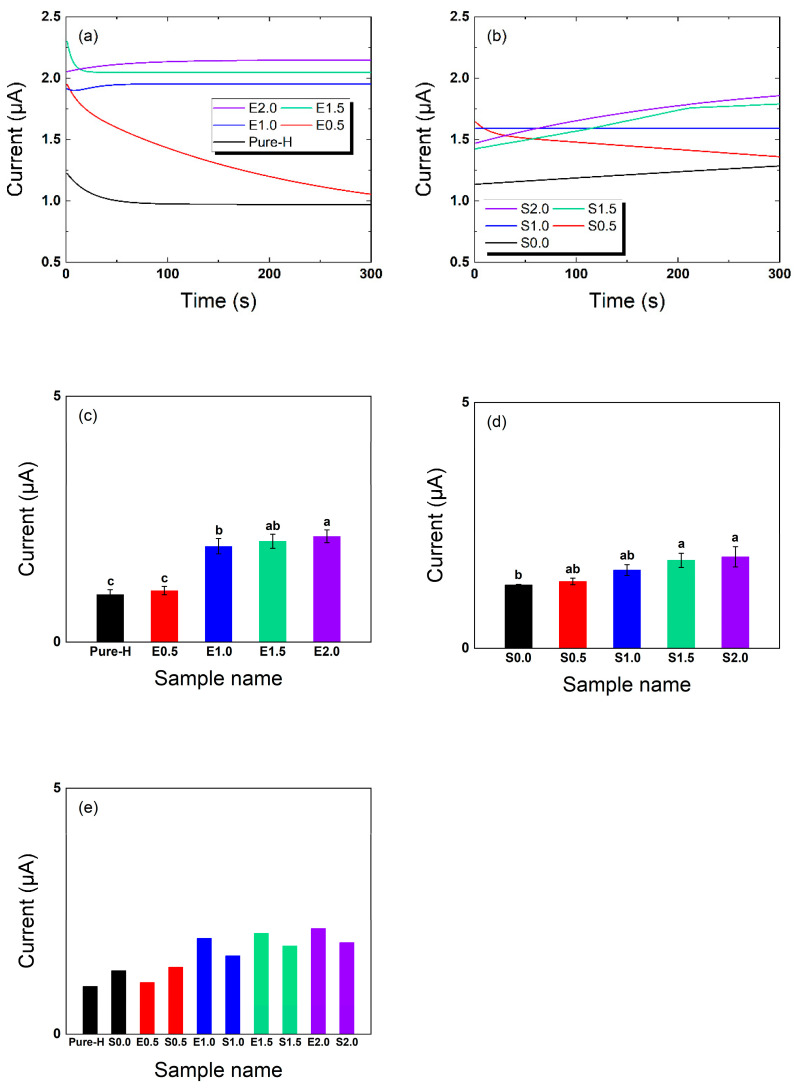
Oxygen transport evaluation measured by oxygen-induced current. (**a**) Oxygen-induced current (μA) over time for the additive-free counterparts (E-series); (**b**) Oxygen-induced current (μA) for the PVP/NVP-containing S-series incorporating the PVP/NVP matrix; (**c**) ANOVA of maximum oxygen-induced current at 300 s for the E-series; (**d**) ANOVA of maximum oxygen-induced current for the S-series; (**e**) Statistical comparison between the E-series and S-series, confirming that TRISS concentration is the primary driver for increased oxygen-induced current. (*n* = 3. Exact F-statistics and *p*-values for the ANOVA and pairwise comparisons are detailed in the Section 2. Different lowercase letters indicate significant differences among groups according to Tukey’s post hoc test following one-way ANOVA in panels (**c**) and (**d**)).

Figure 5 illustrate the oxygen-induced current measured for each formulation. To clearly distinguish the individual effects of the silicone monomer and the hydrophilic matrix, the additive-free TRISS-only formulations without additives (E2.0, as a representative additive-free counterpart) were evaluated alongside the PVP/NVP-containing S-series. In this study, the amperometric current (μA) generated by oxygen reduction is utilized as a relative proxy signal to evaluate the overall oxygen-transport response of the lenses, rather than representing absolute standardized oxygen permeability ($D_{k}$ or $D_{k}/t$) values as specified by ISO standards. Because hydration-induced swelling alters the final hydrated thickness across formulations, calculating $D_{k}$ would introduce systemic inaccuracies [10]. Therefore, the raw current data is reported to maintain experimental transparency and is strictly used for internal formulation-to-formulation comparisons [13].

Increasing the TRISS content substantially improved the oxygen transport capacity of the lenses. For the additive-free formulations, the oxygen-induced current in the absolute control (Pure-H) nearly doubled, increasing from approximately 0.97 μA to 2.15 μA in the 2.0 wt% TRISS formulation (E2.0). All TRISS-containing lenses exhibited significantly higher current values than the silicone-free control group without silicone. This is consistent with the well-established effect of siloxane monomers, such as TRISS, in increasing the oxygen transmission of hydrogels.

In the PVP/NVP-containing S-series, the oxygen-induced current decreased slightly compared to the corresponding additive-free counterparts. For instance, S2.0 showed an average current of 1.86 μA compared with 2.15 μA for E2.0. This modest reduction (approximately 13.5%) suggests that the incorporation of PVP and NVP may slightly attenuate the oxygen-transport response under the present measurement conditions. Since water itself has a lower oxygen diffusion rate than silicone, a slight trade-off exists between increasing surface wettability and maintaining maximum oxygen-induced current.

Nevertheless, all TRISS-containing formulations in the S-series showed higher oxygen-induced current than the silicone-free controls under identical measurement conditions. These data indicate that TRISS incorporation enhances the oxygen-transport response within the present experimental setup, while the PVP/NVP matrix introduces a modest reduction in current in exchange for improved surface wettability and hydration behavior.

ANOVA results (Figure 5c–e) confirmed statistically significant differences were observed (E-series: F(4,10) = 497.45, *p* = 1.87 × 10^−11^; S-series: F(4,10) = 4.26, *p* = 0.02879). For the pairwise matched comparisons in Figure 5e, the addition of the PVP/NVP matrix did not result in statistically significant differences at corresponding TRISS concentrations: Pure-H vs. S0.0 (*p* = 0.31409), E0.5 vs. S0.5 (*p* = 0.33971), E1.0 vs. S1.0 (*p* = 0.17942), E1.5 vs. S1.5 (*p* = 0.56477), and E2.0 vs. S2.0 (*p* = 0.43866). Within the S-series, TRISS-containing formulations (S0.5–S2.0) consistently showed significantly higher oxygen-induced current than the baseline containing exclusively hydrophilic additives (S0.0). These results highlight that while TRISS is the primary factor for improving oxygen delivery, the hydrophilic additives introduce a measurable but acceptable trade-off to ensure physiological comfort.

### 2.6. Mechanical Properties

**Figure 6:**
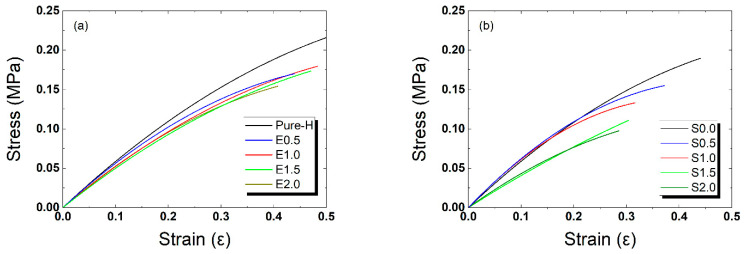
Tensile test results showing the stress–strain behavior. (**a**) Additive-free counterparts (E-series), (**b**) PVP/NVP-containing S-series formulations showing enhanced flexibility (reduced modulus) due to the PVP/NVP matrix.

Figure 6 illustrates the stress–strain behavior of the synthesized silicone hydrogel materials, while Table 3 provides a comprehensive summary of the S-series performance, including its physicochemical properties (EWC, oxygen transport potential), hydration-induced dimensional stability (diameter change), and mechanical properties (Young’s modulus, maximum tensile stress, and elongation at break).

For the PVP/NVP-containing S-series formulations, a progressive decrease in Young’s modulus was observed as the TRISS concentration increased from 0 to 2.0 wt%. Specifically, the Young’s modulus decreased from 0.61 MPa for S0.0 to 0.39 MPa for S2.0, indicating that the incorporation of TRISS enhances the flexibility of the lens matrix. Similarly, the maximum tensile stress and elongation at break exhibited a downward trend with increasing TRISS content, with S2.0 showing the lowest values of 0.10 MPa and 28.58%, respectively.

These results suggest that TRISS incorporation increased the oxygen-induced current, whereas the PVP/NVP matrix improved surface rehydration while shifting the mechanical response toward lower modulus and lower elongation at break at high TRISS loading. However, all S-series formulations maintained sufficient mechanical integrity. Initial expectations suggested that higher TRISS content would increase material stiffness and decrease elongation at break, while NVP/PVP would soften the lens. The results were only partially consistent with these trends, because the Young’s modulus exhibited limited variation within the tested TRISS range (0–2.0 wt%). In the additive-free formulations, the Young’s modulus decreased slightly from 0.62 MPa for Pure-H to 0.50 MPa for E2.0. This indicates that the modulus change remained limited within the tested TRISS range.

However, TRISS had a clearer impact on extensibility. The elongation at break decreased from 51.82% in Pure-H to 40.64% in E2.0 as the TRISS concentration increased, indicating reduced stretchability at higher TRISS levels. In the PVP/NVP-containing S-series, the modulus for S2.0 was 0.39 MPa, lower than that of E2.0 (0.50 MPa), while the elongation at break of S2.0 was 28.58%, also lower than that of E2.0 (40.64%). Thus, within the present formulation window, the PVP/NVP matrix was associated with a softer but less extensible mechanical profile at 2.0 wt% TRISS.

Overall, the moduli of all formulations remained within the range of 0.39–0.61 MPa, which is comparable to many commercial soft contact lenses. The primary distinction lay in toughness; high-TRISS formulations exhibited lower ductility, whereas low-TRISS or additive-free hydrogels demonstrated higher elongation before failure. The findings suggest that incorporating hydrophilic additives at a moderate TRISS concentration (approximately 1.0 wt%, as seen in S1.0) provides a balanced formulation of mechanical properties. Such formulations are sufficiently rigid for shape retention and handling, yet flexible enough to conform comfortably to the ocular surface. Within the tested formulation window, the fixed PVP/NVP matrix (1.0/0.5 wt%) enables 2.0 wt% TRISS loading while preserving wettability and reducing Young’s modulus relative to the additive-free comparator.

## 3. Conclusions

This study established a silicone hydrogel contact lens platform by integrating a low-concentration 3-[tris(trimethylsilyloxy)silyl]propyl methacrylate (TRISS) monomer with a dual-hydrophilic polyvinylpyrrolidone (PVP) and N-vinyl-2-pyrrolidone (NVP) matrix. Our comprehensive evaluation confirmed that the fixed PVP/NVP system (1.0/0.5 wt%) provides a workable design window for incorporating TRISS up to 2.0 wt% without the catastrophic loss of wettability observed in the additive-free comparator. Notably, the S2.0 formulation achieved an approximately 1.9-fold increase in oxygen-induced current relative to Pure-H (0.97 μA to 1.86 μA) while maintaining optical transparency above 97%. The efficacy of the PVP/NVP matrix was most clearly demonstrated by the final equilibrium comparison at 2.0 wt% TRISS: the equilibrium contact angle was reduced from 93.79° in E2.0 to 56.04° in S2.0, while the Young’s modulus decreased from 0.50 MPa to 0.39 MPa. These data show that the fixed PVP/NVP matrix mitigates the hydrophobic penalty of TRISS while preserving a high oxygen-related response within the tested low-silicone window.

In conclusion, the strategic combination of TRISS and the PVP/NVP matrix established in this study provides a promising material framework for next-generation contact lenses that simultaneously balances oxygen transport potential, surface wettability, and mechanical flexibility. However, because these findings are derived from in vitro evaluations, further clinical studies are required to assess on-eye performance, including pre-lens tear film break-up time [29] and protein deposition [30]. Moreover, the dimensional stability described in this study is confined to hydration-induced expansion, whereas stability against other environmental factors, such as temperature [31,32] and pH [33], remains to be determined.

Beyond conventional vision correction and dry eye alleviation, the present findings demonstrate that this highly tunable, stable, and transparent hydrogel platform directly addresses critical material-level bottlenecks of wearable smart lenses. By successfully mitigating the hydrophobic persistence of silicone and strictly suppressing excessive hydration-induced swelling, this substrate effectively minimizes the risks of sensor delamination and geometric warpage of micro-structures. Therefore, it serves as a highly viable foundational matrix for future smart contact lens systems incorporating ocular drug-delivery components [20], microfluidic architectures [21], theranostic applications [22], tear-glucose biosensors [23], and intraocular pressure monitoring modules [24].

## 4. Materials and Methods

### 4.1. Materials

The materials used in this study were strategically selected to balance the hydration-induced dimensional stability, oxygen transport potential, and surface hydrophilicity of the contact lenses. The primary polymer matrix consists of a silicone hydrogel system, which incorporates specific hydrophilic additives designed to effectively modulate the inherent hydrophobicity of the silicone components.

#### 4.1.1. Chemical Reagents

The base monomeric system utilized 2-hydroxyethyl methacrylate (HEMA) as the primary hydrophilic monomer. To ensure the structural integrity and functional performance of the polymer matrix, several specialized agents were employed: ethylene glycol dimethacrylate (EGDMA) as a cross-linking agent, glycidyl methacrylate (GMA) for enhanced mechanical strength, and methacrylic acid (MA) to further improve surface wettability.

The silicone functionality was provided by 3-[tris(trimethylsilyloxy)silyl]propyl methacrylate (TRISS), which serves as the key component for achieving high oxygen transport capacity and enhanced hydration-induced dimensional stability. To balance the hydrophobic nature of TRISS, a complementary hydrophilic additive system was integrated, consisting of N-vinyl-2-pyrrolidone (NVP) and polyvinylpyrrolidone (PVP). In this system, NVP contributes to water retention and surface wettability, while PVP improves overall bulk hydrophilicity and biocompatibility. All reagents, including the initiators, were sourced from Sigma-Aldrich (St. Louis, MO, USA), with the exception of the thermal initiator 2,2′-azobis(2,4-dimethylvaleronitrile) (ADVN), which was obtained from Wako Pure Chemical Industries, Ltd. (Osaka, Japan).

#### 4.1.2. Equipment and Instrumentation

The polymerization and mixing processes were conducted using a high-viscosity digital stirrer (SHT-50DX, DAIHAN Scientific Co., Ltd., Wonju-si, Gangwon-do, Republic of Korea) to ensure uniform monomer distribution, complemented by a rotary evaporator (GM-0.50II, Faithful Instrument (Hebei) Co., Ltd., Huanghua, Cangzhou, Hebei Province, China) and a precision heating/cooling circulator (SCR-12, DAIHAN Scientific Co., Ltd., Wonju-si, Gangwon-do, Republic of Korea) for controlled thermal management. For the fabrication of the contact lenses, custom-designed injection-molded polypropylene molds were utilized, supported by an air compressor system to effectively manage the flow of the viscous polymer mixtures.

Comprehensive characterization of the fabricated lenses was enabled through a specialized suite of analytical instruments. A UV–Vis spectrophotometer (Evolution 201, Thermo Fisher Scientific, Waltham, MA, USA) was employed to verify optical transmittance, while surface wettability was quantified using a contact angle goniometer (Phoenix 300T, Surface Electro Optics Co., Ltd., Suwon-si, Gyeonggi-do, Republic of Korea). The physiological performance of the lenses was further evaluated using an oxygen permeability analyzer (201T Permeometer™, Createch Rehder Development Company, Greenville, SC, USA) to measure the oxygen-induced amperometric current as a relative proxy for oxygen transport potential, and their structural integrity was assessed via a universal tensile testing machine (Lloyd LRX-plus, Lloyd Instruments, AMETEK, Bognor Regis, West Sussex, UK). This integrated equipment set provided precise control over synthesis conditions and ensured a thorough evaluation of the material properties.

### 4.2. Method

#### 4.2.1. Design of Contact Lens

**Figure A3:**
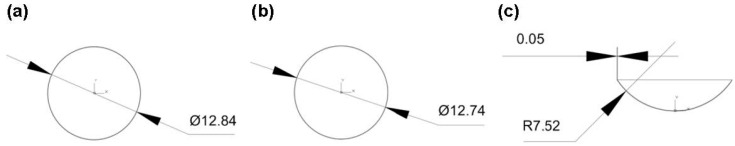
Geometric design and structural specifications of the fabricated contact lens. (**a**) Frontal view illustrating the total outer diameter of 12.84 mm; (**b**) Frontal view of the inner optic zone diameter specified at 12.74 mm; (**c**) Cross-sectional side view highlighting the central thickness of 0.05 mm and the base curve radius (R) of 7.52 mm. The design ensures an optimal corneal fit and minimizes hydration-induced flexure.

The contact lenses were designed using AutoCAD 2023 (Autodesk, San Rafael, CA, USA) to ensure precise geometry and consistent optical performance, as illustrated in the front and side views of the lens model in Figure A3. The key design parameters were strategically selected to ensure a proper corneal fit and to minimize lens flexure during the hydration process; these included a base curve radius of 7.52 mm, a central thickness of 0.05 mm, and an outer diameter of 12.84 mm with an inner optic zone diameter of 12.74 mm.

The structural design of the lens incorporated a controlled fraction of TRISS to achieve a functional balance between mechanical rigidity and flexibility. Furthermore, to address the inherent surface hydrophobicity—a well-known limitation of silicone-based materials—a complementary system of hydrophilic additives [16,18], specifically PVP and NVP, was integrated into the design. This approach was intended to enhance surface wettability while preserving the structural and optical advantages of the silicone hydrogel matrix.

#### 4.2.2. Polymer Preparation

The polymer matrix for the contact lenses was synthesized via a controlled free-radical polymerization process, with the primary objective of calibrating the balance between oxygen transport potential, hydration-induced dimensional stability, and surface wettability. All chemical components—comprising the base monomers (HEMA, EGDMA, GMA, and MA) and the functional additives (TRISS, NVP, and PVP)—were weighed according to the predefined percentages detailed in the formulations. These components were introduced into a 500 mL glass reactor and subjected to continuous stirring at 500 rpm under a vacuum at 25 °C for 3 h. This extended mixing period, utilizing a high-viscosity digital stirrer, was critical to ensuring both thorough homogenization and complete degassing of the solution. During the final 30 min of this process, the thermal initiator ADVN (0.300–0.316 wt%) was introduced under sustained stirring to facilitate uniform initiator distribution, thereby promoting a consistent polymerization reaction upon subsequent heating.

In total, 10 distinct solutions were prepared to systematically evaluate the influence of TRISS concentration and the hydrophilic matrix on lens performance. As established in the Unified Formulation Design (see Table 1 in Section 2), these solutions were categorized to reflect their strategic roles in the study. The PVP/NVP-containing S-series represents the core experimental group, consisting of a fixed hydrophilic matrix (NVP/PVP at a 0.5/1.0 wt% ratio) with TRISS concentrations incrementally increased from 0 to 2.0 wt% the defined hydrophobic threshold intended to prevent optical deterioration and macro-phase separation [16,17] to validate the performance of the system. To isolate and verify the specific effects of the additives, a parallel set of additive-free counterparts was prepared with identical TRISS concentrations excluding the PVP/NVP matrix. Additionally, a pure hydrogel formulation, designated as Pure-H, was utilized as the absolute control to benchmark all physical and chemical property improvements.

#### 4.2.3. Contact Lens Fabrication

The polymerized material was molded into contact lenses through a precision injection-molding process followed by controlled thermal curing to ensure structural integrity. This fabrication pathway was specifically designed to maintain the intended optical and mechanical properties of the material while providing precise control over the dimensional stability and shape of the lens.

##### Mold Design and Preparation

All molds were designed to be an inverted replica of the lens geometry so that the intended lens shape is accurately produced during the injection molding process. The molds utilized for lens casting were manufactured via a dedicated mold-casting technique. To facilitate efficient removal of the polymerized lens, the male and female molds were fabricated from different polymeric materials with specific release characteristics: the male mold was made of polybutylene terephthalate (PBT, Celanese Corporation, Irving, TX, USA), while the female mold consisted of polyethylene (PE, LyondellBasell, Houston, TX, USA).

##### Contact Lens Specimen Preparation

**Figure 7:**
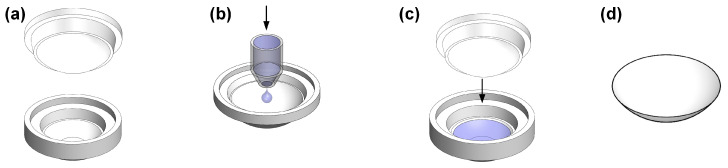
Schematic representation of the contact lens fabrication process via cast-molding. (**a**) Preparation of the precision-machined male (PBT) and female (PE) molds; (**b**) Injection of 200 μL of the degassed pre-polymer monomer solution into the female mold; (**c**) Secure assembly of the male and female molds to define the lens geometry; (**d**) Separation of the polymerized lens following the controlled thermal curing cycle (127 °C for 20 min). The arrows in panels (**b**) and (**c**) indicate the direction of pre-polymer injection and mold assembly, respectively.

The manufacturing process for the contact lens specimens, utilizing the prepared pre-polymer solution and reverse-designed plastic molds, is illustrated in Figure 7. For each lens, 200 μL of the mixed pre-polymer solution was precisely injected into the pre-designed, lens-shaped female mold, which was then securely joined with the corresponding male mold.

To ensure the stable initiation of free-radical polymerization while mitigating issues such as premature gelation or thermal shock, the assembled molds were placed in a controlled oven. The temperature was gradually increased to 127 °C and maintained for 20 min to achieve complete polymerization of the material. Following the curing process, the specimens were slowly cooled to room temperature to minimize internal stresses. Finally, the polymerized lenses were carefully separated from the molds for subsequent characterization.

##### Fabrication Quality Control

To ensure consistency across the various formulations, several systematic quality control (QC) checks were performed on the polymerized specimens. The center thickness and overall shape stability of the lenses were measured using a specialized lens analysis system (JCF/F, Optimec Metrology Ltd., Malvern, Worcestershire, UK). Furthermore, the surface uniformity and structural integrity of each lens were inspected under optical microscopy (KH-7700, Hirox Co., Ltd., Tokyo, Japan) to identify and exclude any specimens exhibiting physical defects or surface irregularities.

#### 4.2.4. Optical and Structural Analysis

All fabricated specimens were systematically measured and analyzed to evaluate group-specific characteristics, including light transmittance, hydration-induced water content, surface wettability, oxygen-induced current, and mechanical properties. These evaluations were conducted to assess the effects of TRISS incorporation under the fixed PVP/NVP condition within the S-series framework.

##### Light Transmittance

Before analysis, all specimens were hydrated in phosphate-buffered saline (PBS) for 48 h to reach equilibrium. To maintain this hydration state during testing, each specimen was placed in a cuvette containing PBS (BR759125, BRAND GmbH + Co. KG, Wertheim, Germany), and the spectral transmittance across the visible range (380–780 nm) was recorded at a 1.0 nm resolution using a UV-Vis spectrophotometer. According to ISO 18369-3:2017 standards [34], a minimum transmittance of 90% was established as the threshold for optical acceptability in contact lens applications.

##### Hydration-Induced Diameter Change

To assess hydration-induced diameter change, the expansion behavior of the lenses during hydration was monitored. Dry specimens were placed in phosphate-buffered saline (PBS) at room temperature (pH 7.5), and their diameters were measured at one-minute intervals for a duration of 15 min [34,35]. The resulting diameter-change curves were fitted using the exponential approach-to-equilibrium model in Equation (1).

(1) $$∆D(t)=A_{D}\left[1-exp\left(-\frac{t}{\tau_{D}}\right)\right]$$

where $\Delta D\left(t\right)$ is the diameter change at time t, $A_{D}$ is the fitted asymptotic maximum diameter change, and $\tau_{D}$ is the fitted time constant for hydration-induced dimensional expansion. This analysis reflects the dimensional response observed during the transition from the dry state to the hydrated state under the present test conditions. However, resistance to other environmental stressors, such as variations in temperature, pH, or dehydration, was not evaluated in this study and remains a subject for future investigation [31,32,33]. The fitted kinetic parameters for both the E-series and S-series were summarized in Table 2.

##### Water Content

The equilibrium water content (EWC) of the fabricated lenses was determined in accordance with the ISO 18369-4:2017 standard. To obtain the dry weight ($W_{d}$), the lenses were first dried in a forced-air oven (OF-02, JEIO TECH Co., Ltd., Daejeon, Republic of Korea) until a constant mass was reached. The specimens were then fully hydrated in phosphate-buffered saline (PBS) to measure their saturated swelling weight ($W_{s}$).

The EWC was calculated using Equation (2) and compared across the different formulation groups to evaluate the impact of TRISS and hydrophilic additives on the material’s water-retention capacity [13].

(2) $$EWC\left(\%\right)=\left(\frac{W_{s}-W_{d}}{W_{s}}\right)\times 100$$

For kinetic analysis, the time-dependent EWC uptake curves were fitted using the exponential approach-to-equilibrium model in Equation (3).

(3) $$EWC(t)=A_{EWC}\left[1-exp\left(-\frac{t}{\tau_{EWC}}\right)\right]$$

where $EWC\left(t\right)$ is the water content at time t, $A_{EWC}$ is the fitted asymptotic equilibrium water content, and $\tau_{EWC}$ is the fitted time constant for bulk water uptake.

##### Wettability

The surface wettability of the fabricated lenses was evaluated through static contact angle measurements using the sessile drop method. All specimens were fully hydrated in phosphate-buffered saline (PBS) for 48 h to ensure equilibrium before testing. An 8 μL droplet of deionized water was precisely placed on the center of each specimen surface, and the contact angle was recorded at specific time intervals of 0, 0.17 (10 s), 1, 5, 10, and 15 min to monitor the time-dependent wetting behavior. This time-series analysis was instrumental in calculating the hydration time constant ($\tau_{CA}$), providing a quantitative measure of the surface’s transition to a hydrophilic state.

To quantitatively analyze the surface rehydration characteristics, the changes in contact angle (θ) over time (t) were modeled using the following exponential decay equation:

(4) $$\;\theta \left(t\right)=\theta_{end}+\left(\theta_{0}-\theta_{end}\right)exp\left(-\frac{t}{\tau_{CA}}\right)\;$$

where θ(t) is the contact angle at time t, $\theta_{0}$ is the initial contact angle, $\theta_{end}$ is the fitted terminal contact angle within the observation window, and $\tau_{CA}$ is the fitted time constant for in vitro surface wetting recovery. This parameter was used only for contact-angle decay analysis and was not conflated with the kinetic parameters derived from diameter change or bulk water uptake.

##### Oxygen-Induced Current Measurement

The oxygen-induced current of the lenses was measured using a polarographic oxygen sensor (201T Permeometer™, Createch Rehder Development Company, Greenville, SC, USA) [13] in accordance with established protocols. To mimic physiological ocular conditions, each specimen was placed in the sensor chamber in direct contact with a cathode electrode at a controlled temperature of 35 °C and a relative humidity of approximately 98%. The current response across each specimen was monitored for 300 s. Because direct thickness values corresponding to the oxygen-measurement state were unavailable, the recorded current signals were used as relative proxy signals for formulation-to-formulation comparison under identical measurement settings. To ensure strict comparative consistency, the steady-state oxygen-induced current (μA) for each formulation was defined as the current value recorded at the 300-s mark.

##### Mechanical Property Evaluation

To evaluate the mechanical characteristics of the materials fabricated with varying monomer concentrations, tensile tests were performed to measure the Young’s modulus, maximum tensile stress, and elongation at break [8]. For specimen preparation, the polymer mixtures were coated onto a substrate and cured. The resulting films were then immersed in deionized water to achieve full hydration, after which all specimens were precisely cut into uniform rectangular strips using a precision punching machine (QM130, QMESYS Corp., Uiwang-si, Gyeonggi-do, Republic of Korea). The standardized dimensions of each tensile specimen were 130 mm in length, 1.3 mm in width, and 0.65 mm in thickness. The mechanical properties were determined using a universal testing machine (Lloyd LRX-plus, Lloyd Instruments, AMETEK, Bognor Regis, West Sussex, UK) in accordance with the ASTM D412 standard [14]. Each sample was subjected to a constant stretching rate of 10 mm/min until failure occurred, and tensile testing was performed on 10 specimens for each formulation to ensure statistical reliability.

From the resulting stress-strain curves, the Young’s modulus was calculated from the slope of the initial elastic region, while the maximum tensile stress and elongation at break were recorded at the point of fracture. These data provided a comprehensive assessment of how the interplay between the silicone monomer and hydrophilic additives influences the structural integrity and flexibility of the lens matrix.

### 4.3. Statistical Analysis

All experimental data are presented as the mean ± standard error (SE). Statistical analyses were performed using OriginPro 2024 (64-bit), Version 10.1.0.170 (Student Version) (OriginLab Corporation, Northampton, MA, USA). Statistical comparisons among multiple groups (e.g., within the E-series or S-series) were evaluated using one-way analysis of variance (ANOVA) followed by Tukey’s post hoc test. Direct pairwise comparisons between matched non-additive and PVP/NVP-containing formulations at the same TRISS concentration were evaluated using independent two-sample t-tests. Exact F-statistics, degrees of freedom, and specific *p*-values are detailed comprehensively within the respective Section 2. For all analyses, *p*-values less than 0.05 were considered to indicate statistically significant differences. Sample sizes varied by assay type: To account for batch-to-batch variability, three entirely independent fabrication batches were prepared for each formulation. Hydration-related measurements, wettability, and oxygen-induced current were analyzed with *n* = 3 independent specimens per formulation (one lens randomly selected from each independent batch). Tensile testing was performed with *n* = 10 specimens per formulation. (randomly pooled from the three independent batches).

## Figures and Tables

**Table 1:** Formulations of the pHEMA-based contact lenses with varying TRISS concentrations and the presence of the PVP/NVP matrix.

Group	Sample Name	TRISS (wt%)	PVP/NVP (wt%)	Logic Role
Control	Pure-H	0.00	0.0/0.0	Absolute control
Additive-free Series	E0.5	0.5	0.0/0.0	Additive-free matched comparator
	E1.0	1.0	0.0/0.0	
	E1.5	1.5	0.0/0.0	
	E2.0	2.0	0.0/0.0	
PVP/NVP-containing S-series	S0.0	0.00	1.0/0.5	Hydrophilic baseline
	S0.5	0.50	1.0/0.5	System validation
	S1.0	1.00	1.0/0.5	
	S1.5	1.50	1.0/0.5	
	S2.0	2.00	1.0/0.5	

Note: All formulations were prepared using 2-hydroxyethyl methacrylate (HEMA) as the base hydrophilic monomer. Common supporting components—including EGDMA (cross-linker), GMA and MA (mechanical and hydrophilic reinforcement), and ADVN (thermal initiator)—were maintained within narrow normalized ranges across the formulation set and were not treated as independent design variables. The PVP/NVP matrix consists of a 2:1 ratio of PVP (1.0 wt%) and NVP (0.5 wt%).

**Table 2:** Fitted kinetic and terminal parameters for hydration-induced diameter expansion ($\tau_{D}$, $A_{D}$), equilibrium water content uptake ($\tau_{EWC}$, $A_{EWC}$), and contact-angle decay ($\tau_{CA}$, $\theta_{end}$) for Pure-H, the E-series, and the S-series.

**Sample Name**	Diameter			EWC			Contact-Angle		
	$\tau_{D}$ (min)	$A_{D}$ (%)	$R^{2}$	$\tau_{EWC}$ (min)	$A_{EWC}$ (%)	$R^{2}$	$\tau_{CA}$ (min)	$\theta_{end}$ (°)	$R^{2}$
Pure-H	3.26	16.75	0.996	1.58	35.58	0.994	4.89	35.61	0.756
E0.5	1.68	16.02	0.993	1.83	34.84	0.870	6.27	53.60	0.887
E1.0	1.87	15.28	0.994	1.30	34.57	0.961	2.64	71.30	0.891
E1.5	1.62	15.05	0.992	1.09	31.53	0.961	4.26	72.14	0.964
E2.0	1.68	14.32	0.993	0.83	30.69	1.000	0.81	93.79	1.000
S0.0	3.46	18.70	0.999	2.38	37.81	0.936	8.84	26.92	0.921
S0.5	2.05	16.57	0.995	2.81	37.76	0.778	9.01	36.92	0.940
S1.0	2.19	16.17	0.996	1.52	35.00	0.956	9.18	50.37	0.934
S1.5	2.74	15.22	0.996	1.78	32.35	0.914	6.72	56.04	0.976
S2.0	2.45	15.15	0.997	1.22	31.06	0.960	7.52	56.04	0.960

**Table 3:** Comprehensive physicochemical, physical, and mechanical properties of the fabricated contact lenses.

Sample Name	Physicochemical Property		Physical Property	Mechanical Property		
	EWC (%)	Oxygen-Induced Current (μA)	Hydration-Induced Maximum Diameter Change (%)	Young’s Modulus (MPa)	Maximum Stress (MPa)	Elongation at Break (%)
Pure-H	37.2	0.97	16.7	0.62	0.22	51.82
S0.0	39.1	1.29	18.3	0.61	0.19	45.88
S0.5	37.5	1.36	16.2	0.61	0.16	38.52
S1.0	35.8	1.59	15.8	0.59	0.14	32.13
S1.5	34.2	1.79	15.2	0.42	0.11	30.38
S2.0	33.1	1.86	15.1	0.39	0.10	28.58
E2.0	32.0	2.15	14.2	0.50	0.15	40.64

Note: All data are expressed as mean ± standard error (SE). *n* = 3 for Equilibrium Water Content (EWC), Oxygen-induced Current, and Maximum diameter change; *n* = 10 for mechanical properties (Young’s Modulus, Maximum stress, and Elongation at break). Statistical comparisons were performed as described in Section 4.3, and exact *p*-values are reported in the corresponding Section 2.

**Figure A2:**
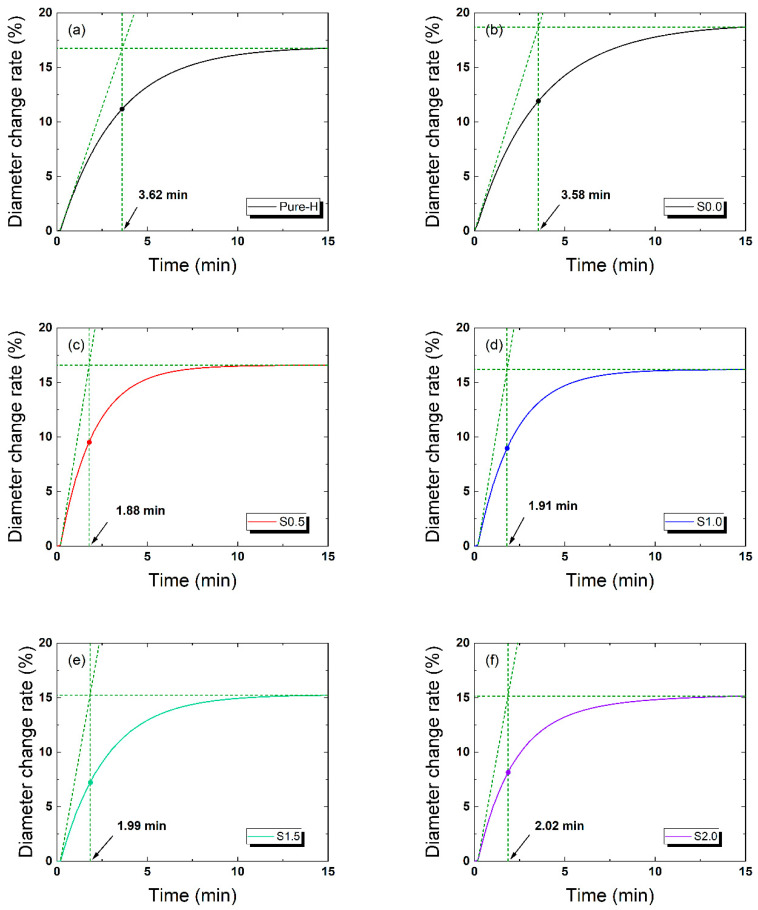
Non-linear fitting of hydration-induced diameter-change saturation for formulations Pure-H to S2.0. The diameter time constant ($\tau_{D}$) was derived from the exponential function Equation (1) for each formulation. The illustrate the fitted dimensional-expansion behavior used for the $\tau_{D}$ analysis summarized in Table 2. The dashed lines represent the non-linear fitted curves obtained using Equation (1). (**a**) Pure-H, (**b**) S0.0, (**c**) S0.5, (**d**) S1.0, (**e**) S1.5, (**f**) S2.0.

## Data Availability

The data presented in this study are available within the article.

## Abbreviations

The following abbreviations are used in this manuscript:

TRISS	3-[tris(trimethylsilyloxy)silyl]propyl methacrylate
PVP	Polyvinylpyrrolidone
NVP	N-vinyl-2-pyrrolidone
EGDMA	Ethylene glycol dimethacrylate
GMA	Glycidyl methacrylate
MA	Methacrylic acid
ADVN	2,2′-Azobis(2,4-dimethylvaleronitrile)
PBS	Phosphate Buffered Saline
QC	Quality Control
UV–Vis	Ultraviolet–Visible Spectrophotometer
ISO	International Organization for Standardization
ASTM	American Society for Testing and Materials

## Appendix A

**Table A1:** Summary of physical properties for non-additive silicone hydrogels (Group 1: Pure-H, E0.5, E1.0, E1.5, and E2.0). The reported oxygen-induced current values represent raw polarographic sensor outputs used for relative comparison among formulations.

Sample Name	TRISS (wt%)	Transmittance (%)	Maximum Diameter Change (%)	Contact Angle (°)	Oxygen-Induced Current (μA)
Pure-H	0.00	99.3	16.7	53.9	0.97
E0.5	0.50	98.8	15.9	71.2	1.05
E1.0	1.00	98.4	15.2	80.1	1.95
E1.5	1.50	97.9	14.8	83.5	2.05
E2.0	2.00	97.3	14.2	95.4	2.15

Note: Oxygen-induced current values correspond to the current measured at 300 s under identical polarographic measurement conditions.

**Table A2:** Chemical reagents and their respective functions in the silicone hydrogel system. The base hydrogel consists primarily of HEMA with small amounts of crosslinker (EGDMA), mechanical reinforcement (GMA), and a wettability monomer (MA). TRISS is the silicone monomer contributing to oxygen transport. NVP and PVP are hydrophilic additives. ADVN is the initiator. Ranges indicate the percentage (by weight) used across the different formulations tested. The narrow ranges reported for EGDMA, GMA, MA, and ADVN reflect normalized composition values across the formulation set and correspond to the actual synthesized batches, with these components treated as supporting formulation components rather than independent design variables.

Component	Function	Amount (%)
2-Hydroxyethyl methacrylate (HEMA)	Base hydrophilic monomer	93.77–98.76
Ethylene glycol dimethacrylate (EGDMA)	Cross-linking agent	0.656–0.691
Glycidyl methacrylate (GMA)	Reinforce mechanical strength	0.188–0.198
Methacrylic acid (MA)	Enhance surface hydrophilicity	0.028–0.030
3-[tris(trimethylsilyloxy)silyl]propyl methacrylate (TRISS)	Silicone monomer	0–2.315
N-vinyl-2-pyrrolidone (NVP)	Hydrophilic additive	0–0.959
Polyvinylpyrrolidone (PVP)	Hydrophilic additive	0–1.918
2,2′-Azobis(2,4-dimethylvaleronitrile) (ADVN)	Polymerization initiator	0.300–0.316
